# AI-enabled D-dimeromics in precision breast oncology: a transformative framework for the identification, stratification, and prognostication of ultra-high-risk disease phenotypes

**DOI:** 10.3389/fonc.2026.1910821

**Published:** 2026-09-01

**Authors:** Emmanuel Ifeanyi Obeagu

**Affiliations:** 1 Division of Haematology, Department of Biomedical and Laboratory Science, Africa University, Mutare, Zimbabwe; 2 Department of Molecular Medicine and Haematology, Faculty of Health Sciences, University of the Witwatersrand, Johannesburg, South Africa

**Keywords:** artificial intelligence, breast cancer, D-dimeromics, oncology, thromboinflammation

## Abstract

Breast cancer is a highly diverse ailment marked by various molecular subtypes, differing clinical paths, and unique treatment reactions. Notwithstanding considerable progress in precision oncology, the prompt detection of patients with ultra-high-risk breast cancer phenotypes continues to be a significant clinical hurdle. Growing evidence suggests that hypercoagulability linked to cancer and thromboinflammation are vital in tumour advancement, metastatic spread, evasion of immunity, and resistance to treatment. D-dimer has become a notable biomarker for coagulation, signalling tumour aggressiveness, disease extent, and unfavorable clinical results. Nonetheless, traditional D-dimer evaluation depends on singular assessments that do not reflect the intricate biological interactions influencing breast cancer advancement. To overcome this limitation, the idea of D-dimeromics has arisen as a comprehensive framework that combines D-dimer with additional haemostatic, inflammatory, molecular, radiological, and clinical data. Simultaneously, progress in artificial intelligence (AI), particularly in machine learning and deep learning, has facilitated the examination of high-dimensional biomedical data and the identification of predictive patterns that exceed the capabilities of conventional statistical methods. This narrative review examines the biological justifications, technological bases, and clinical uses of AI-driven D-dimeromics as a groundbreaking precision oncology approach for detecting ultra-high-risk breast cancer phenotypes. The article examined the connections between coagulation activation and tumour development, the prognostic value of D-dimer in various breast cancer subtypes, and the function of AI in combining multidimensional data for dynamic risk assessment, metastatic prediction, treatment response evaluation, and individualized treatment strategies.

## Introduction

Breast cancer continues to be the most commonly diagnosed cancer and one of the primary causes of cancer-related deaths amongst women globally, representing a significant global health challenge despite significant progress in screening, molecular diagnostics, and treatment options. The shift from traditional treatment methods to precision oncology has notably enhanced patient outcomes by integrating molecular subtyping, genomic profiling, and targeted therapies. Despite this, breast cancer remains highly biologically diverse, as patients with comparable clinicopathological features frequently undergo significantly distinct disease paths. This variation highlights the necessity for more advanced predictive models that can pinpoint patients susceptible to severe disease progression, early relapse, metastatic spread, and treatment ineffectiveness (1, 2). Historically, the assessment of breast cancer risk has depended on elements like tumour dimensions, lymph node involvement, histological grading, hormone receptor levels, HER2 status, Ki-67 proliferation rate, and multigene expression tests. Although these parameters offer useful prognostic insights, they fail to entirely represent the dynamic interplay between tumour biology and systemic host reactions that influence disease advancement. Growing evidence indicates that thrombosis and thromboinflammation related to cancer are not just secondary effects of the disease, but rather active contributors to tumour initiation, progression, angiogenesis, immune modulation, and metastasis. These results have sparked increased interest in coagulation-related biomarkers as possible means for enhancing prognostic evaluations and directing personalized treatment approaches (3, 4).

Of these biomarkers, D-dimer has proven to be one of the most clinically available and biologically significant signs of cancer-related coagulation activation. D-dimer, as a breakdown item of cross-linked fibrin, indicates active thrombin production, fibrin creation, and fibrinolysis. Increased D-dimer levels have been reliably linked to more advanced tumour stages, lymph node spread, distant organ involvement, heightened recurrence risk, and lower survival rates in breast cancer patients. In addition to its conventional use in diagnosing venous thromboembolism, D-dimer is increasingly acknowledged as an indicative marker of the intricate relationship between coagulation, inflammation, endothelial dysfunction, and tumour aggressiveness. Nonetheless, the clinical usefulness of D-dimer has been restricted by its traditional view as a single, static lab measurement, which does not reflect the complex biological mechanisms driving cancer advancement (5, 6). The rise of systems biology and multi-omics technologies has revolutionized the comprehension of cancer as a dynamic ecosystem characterized by complex interactions amongst tumour cells, immune elements, vascular networks, metabolic pathways, and host inflammatory responses. In this context, the idea of D-dimer omics signifies a new approach that goes beyond singular D-dimer assessments to include an expansive thromboinflammatory framework that incorporates coagulation indicators, inflammatory substances, molecular changes, imaging properties, and clinical factors. D-dimeromics, by analysing D-dimer in a wider biological context, provides a chance to gain more profound understanding of disease dynamics and uncover clinically significant phenotypes that might not be visible through standard biomarker evaluation (7–9).

At the same time, artificial intelligence (AI) has become a powerful agent in oncology, facilitating the examination of intricate, high-dimensional datasets that surpass the limits of conventional statistical methods. Algorithms in machine learning, deep learning, and explainable AI can reveal concealed patterns, nonlinear connections, and predictive markers across various data types, such as laboratory biomarkers, radiological images, genomic data, digital pathology, and electronic health records. These technologies have shown impressive promise in cancer detection, prognosis, treatment options, and predicting outcomes. When utilized in D-dimeromics, AI offers a robust analytical framework that can convert a commonly assessed coagulation biomarker into a dynamic source of predictive insight (10–13). The combination of AI and D-dimeromics presents a distinctive chance to recognise ultra-high-risk breast cancer phenotypes marked by increased thromboinflammatory activity, aggressive molecular characteristics, metastatic potential, and unfavorable clinical results. These phenotypes might indicate a group of patients who need closer monitoring, prompt therapeutic action, or customized treatment approaches that exceed conventional care. Additionally, AI-based D-dimeromic models could enable ongoing observation of disease progression, forecasting treatment reactions, and identifying developing resistance, thus promoting a more flexible and personalized strategy for cancer treatment (14–16).

This narrative review explores the changing function of AI-driven D-dimeromics as a revolutionary precision oncology model for detecting ultra-high-risk breast cancer phenotypes. We examine the biological basis connecting coagulation and tumour advancement, assess the prognostic relevance of D-dimer in breast cancer, investigate the concepts and uses of artificial intelligence in biomarker analysis, and underscore the possible clinical consequences, obstacles, and future prospects of this developing area. Integrating thromboinflammatory biology with cutting-edge computational intelligence, AI-driven D-dimeromics could signify a novel advancement in breast cancer risk assessment and tailored medicine.

### Aim of the review

The primary objective of this review is to examine the scientific rationale, technological foundations, and clinical potential of AI-enabled D-dimeromics as a transformative precision oncology strategy for identifying ultra-high-risk breast cancer phenotypes, whilst highlighting current challenges and future opportunities for research and clinical implementation.

## Methods

### Review design and scope

This study was conducted as a narrative review with a structured literature-search approach. A narrative design was selected because the subject of AI-enabled D-dimeromics lies at the intersection of breast oncology, coagulation and fibrinolysis, thromboinflammation, biomarker science, and artificial intelligence (AI), whilst D-dimeromics itself remains an emerging, hypothesis-driven concept without an established terminology or sufficiently mature body of homogeneous studies to support formal meta-analysis. The review therefore aimed to critically synthesize established biological evidence, emerging computational evidence, and adjacent methodological developments relevant to the proposed D-dimeromics framework rather than to estimate a pooled intervention effect or diagnostic accuracy measure.

### Literature search strategy

A structured search of PubMed/MEDLINE, Scopus, Web of Science, and Google Scholar was undertaken to identify relevant peer-reviewed literature. The search covered studies available up to July 2026, with particular emphasis on literature published from 2023 to 2026 to capture recent advances in AI-enabled prediction, cancer-associated thrombosis, longitudinal biomarker modelling, and multi-omics precision oncology. Search terms were used individually and in combinations, including: *breast cancer*, *breast carcinoma*, *D-dimer*, *fibrin degradation products*, *coagulation*, *fibrinolysis*, *thromboinflammation*, *immunothrombosis*, *cancer-associated thrombosis*, *venous thromboembolism*, *prognosis*, *survival*, *recurrence*, *treatment response*, *artificial intelligence*, *machine learning*, *deep learning*, *longitudinal modelling*, *dynamic biomarkers*, *radiomics*, *proteomics*, *metabolomics*, *multi-omics*, and *precision oncology*. Boolean operators (AND, OR) were applied as appropriate to combine concepts relating to breast cancer, D-dimer biology, clinical outcomes, and computational modelling. Because the term “D-dimeromics” is not yet a standardized indexing term and the directly relevant literature is limited, the search deliberately incorporated adjacent evidence from cancer-associated thrombosis, longitudinal D-dimer analysis, AI-based clinical prediction, and multimodal oncology. Reference lists of relevant reviews and key primary studies were also examined to identify additional eligible publications.

### Eligibility criteria

Studies were considered for inclusion when they addressed one or more of the following domains: the biological relationship between malignancy and coagulation or fibrinolysis; D-dimer in breast cancer prognosis, progression, recurrence, metastasis, or thrombosis; AI or machine-learning models relevant to breast cancer risk prediction or cancer-associated VTE; longitudinal or dynamic biomarker modelling; and multimodal or multi-omics approaches relevant to precision breast oncology. Eligible evidence included original clinical studies, prospective and retrospective cohorts, externally validated prediction studies, systematic reviews, meta-analyses, authoritative guidelines, consensus statements, and high-quality mechanistic studies. Recent studies were prioritized for rapidly evolving computational and translational topics, whereas seminal older studies were retained where necessary to establish fundamental biological mechanisms. Studies were excluded or deprioritized when they were unrelated to breast cancer or the conceptual framework, provided insufficient methodological information, duplicated previously published datasets without substantial additional information, consisted solely of non-scholarly commentary, or made claims that could not be adequately evaluated from the available report. Non-breast cancer studies were included selectively when they provided directly transferable methodological or biological evidence concerning cancer-associated thrombosis, D-dimer dynamics, or AI-based longitudinal prediction.

### Study selection and evidence prioritization

Titles and abstracts identified through the searches were assessed for relevance to the predefined conceptual domains. Potentially relevant full-text publications were then evaluated for their contribution to the biological rationale, prognostic evidence, computational methodology, or translational framework of D-dimeromics. As this was a single-author narrative review, literature identification, relevance assessment, and evidence synthesis were conducted by the author. Evidence was prioritized according to methodological relevance and strength. Greater weight was assigned to large prospective or multicentre cohorts, systematic reviews and meta-analyses, externally validated prediction models, studies with clearly defined clinical outcomes, and investigations reporting appropriate measures of discrimination and calibration. For AI studies, particular attention was given to sample size, input features, algorithm selection, comparator models, internal or external validation, and reported performance measures such as the area under the receiver operating characteristic curve, concordance index, sensitivity, specificity, and predictive values. Mechanistic studies were used primarily to establish biological plausibility and were not treated as direct evidence of clinical utility. Similarly, studies of AI-based cancer-associated thrombosis or multimodal oncology were classified as adjacent or enabling evidence when they did not specifically evaluate an AI-enabled D-dimeromics model in breast cancer.

### Evidence synthesis and critical appraisal

The evidence was synthesized narratively according to four major domains: (1) the biological basis of D-dimeromics; (2) D-dimer as a prognostic and thromboinflammatory biomarker in breast cancer; (3) AI and multimodal computational approaches relevant to D-dimeromics; and (4) potential integration with precision breast oncology. The synthesis distinguished between established evidence, emerging empirical evidence, and hypothesis-generating propositions. Particular attention was given to conflicting and negative findings, assay heterogeneity, differences in D-dimer thresholds, variation in outcome definitions, confounding, missing data, model overfitting, algorithmic bias, and limitations in external validation. No formal quantitative evidence-grading system or meta-analysis was undertaken because of the heterogeneity of study designs, endpoints, assays, computational methods, and the emerging nature of the central concept. Nevertheless, the interpretation of evidence was guided by study design, sample size, methodological transparency, adjustment for major confounders, validation strategy, consistency across studies, and directness of relevance to breast cancer.

### Conceptual framework development

**Figure 1:**
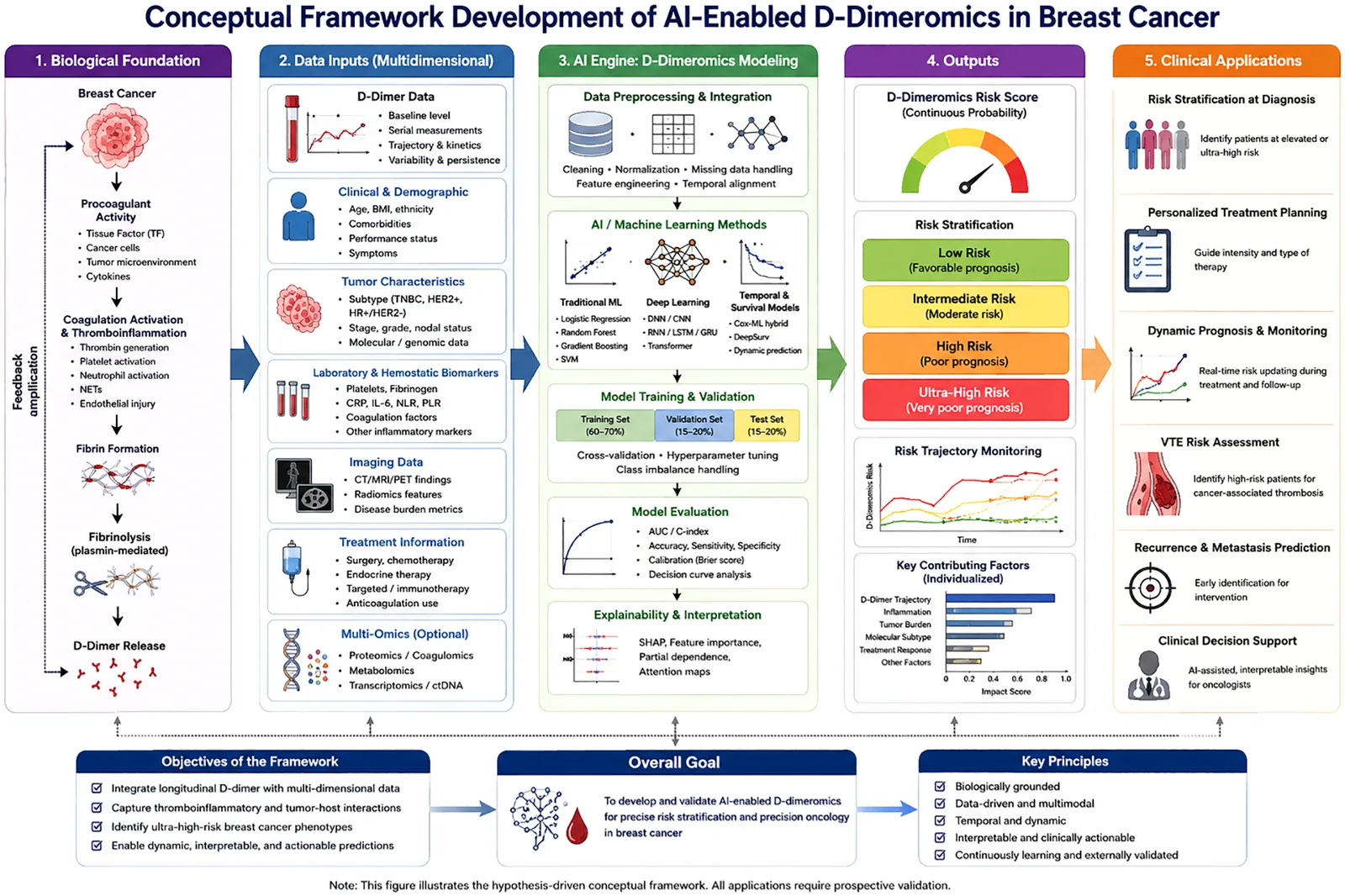
Conceptual framework development.

The proposed AI-enabled D-dimeromics framework was developed through integration of evidence across coagulation biology, fibrinolysis, thromboinflammation, breast cancer prognosis, longitudinal biomarker analysis, and AI-enabled multimodal prediction. D-dimeromics was treated as a hypothesis-driven conceptual framework rather than an established clinical discipline. The framework specifically examined whether the magnitude, trajectory, persistence, variability, and treatment responsiveness of D-dimer could potentially be integrated with clinical, pathological, haemostatic, inflammatory, imaging, treatment, and molecular variables to identify dynamic high-risk phenotypes. Claims concerning clinical implementation were therefore interpreted cautiously and differentiated from proposed future applications requiring prospective validation (**Figure 1**).

### Methodological limitations

The absence of a formal systematic-review protocol, independent dual screening, standardized risk-of-bias assessment, and quantitative evidence grading limits reproducibility. Furthermore, the lack of standardized terminology for D-dimeromics necessitated inclusion of indirect and adjacent evidence. These limitations were considered throughout the interpretation of the literature, and conclusions were framed as hypothesis-generating where direct empirical evidence was insufficient.

### The biological basis of D-dimeromics in breast cancer

The biological rationale for D-dimeromics in breast cancer arises from the close interconnection between malignant progression, coagulation activation, inflammation, platelet biology, endothelial dysfunction, and fibrinolysis. Rather than representing a tumour-specific biomarker, D-dimer is a downstream product of cross-linked fibrin degradation and therefore reflects the net consequence of fibrin formation and subsequent fibrinolytic activity. In breast cancer, these processes may be continuously influenced by tumour burden, metastatic activity, host inflammatory responses, treatment exposure, and cancer-associated thrombosis. Accordingly, the proposed concept of D-dimeromics extends beyond interpretation of a single D-dimer concentration to encompass the multidimensional and longitudinal characterization of D-dimer within the broader tumour–host thromboinflammatory ecosystem (9, 17). Breast cancer cells can promote a procoagulant state through several interconnected mechanisms. Tumour-associated expression of tissue factor, activation of coagulation pathways, release of procoagulant extracellular vesicles, inflammatory signalling, and endothelial perturbation can collectively enhance thrombin generation and fibrin formation. Tissue factor is particularly relevant because activation of the tissue factor–factor VIIa pathway initiates downstream coagulation and promotes thrombin generation. However, the biological importance of coagulation in malignancy extends beyond haemostasis. Thrombin and other coagulation-associated mediators can influence tumour-cell signalling, angiogenesis, stromal interactions, and inflammatory responses. Consequently, increased fibrin turnover may reflect not only clinically apparent thrombosis but also broader tumour-associated activation of the haemostatic system (18–20).

Fibrin provides an important mechanistic bridge between coagulation and malignant progression. Within the tumour microenvironment and circulation, fibrin-rich matrices can contribute to cellular adhesion, extracellular matrix remodelling, and interactions amongst tumour cells, platelets, leukocytes, and endothelial cells. During metastatic dissemination, tumour cells entering the circulation may interact with platelets and fibrin, potentially facilitating vascular survival and metastatic colonization. Subsequent degradation of cross-linked fibrin generates D-dimer, creating a measurable downstream signal of ongoing coagulation and fibrinolytic activity. Persistent or changing D-dimer concentrations may therefore capture aspects of the dynamic biological processes accompanying tumour progression, although such changes remain nonspecific and require careful clinical contextualization (21, 22). Platelets further connect tumour biology with coagulation and inflammation. Tumour-associated platelet activation can facilitate platelet–tumour cell interactions and contribute to the release of growth factors, cytokines, angiogenic mediators, and procoagulant substances. Platelet activation may also amplify thrombin generation and fibrin deposition. These interactions establish a reciprocal relationship in which malignant cells promote haemostatic activation whilst components of the haemostatic system may support tumour progression. Thus, D-dimer can be conceptualized as a downstream indicator of a broader biological network rather than an isolated marker of fibrinolysis (23).

Inflammation adds another layer of complexity. Breast cancer can induce systemic and local inflammatory responses involving cytokines, neutrophils, monocytes, platelets, and endothelial cells. Pro-inflammatory signalling may enhance tissue factor expression, activate endothelial cells, promote platelet reactivity, and suppress physiological anticoagulant mechanisms. Conversely, coagulation proteases can activate cellular signalling pathways that reinforce inflammatory responses. This bidirectional interaction between inflammation and coagulation constitutes a thromboinflammatory axis that may contribute to cancer progression and cancer-associated thrombosis (24). Immunothrombosis may also contribute to this biological continuum. Activated neutrophils and other innate immune mechanisms can interact with platelets and coagulation pathways, creating localized prothrombotic environments. These processes may increase fibrin formation and turnover whilst simultaneously influencing tumour–immune interactions. The convergence of inflammation, immune activation, endothelial perturbation, platelet activity, and coagulation therefore provides a mechanistic basis for considering D-dimer as a composite downstream signal of tumour–host interactions (25). The biological pathway underlying the proposed D-dimeromics framework can consequently be conceptualized as follows: Breast cancer biology and tumour burden → tissue factor and procoagulant signalling → platelet, endothelial, and inflammatory activation → thrombin generation → cross-linked fibrin formation → fibrinolysis → circulating D-dimer This pathway is neither linear nor tumour-specific. Infection, increasing age, renal or hepatic dysfunction, recent surgery, hospitalization, trauma, pregnancy, cardiovascular disease, and venous thromboembolism can independently influence D-dimer concentrations. Treatment itself may also alter D-dimer dynamics through tumour response, tissue injury, inflammation, vascular effects, or thrombotic complications. These factors constitute essential confounders and contextual variables for any D-dimeromic model (26).

The central hypothesis of D-dimeromics is therefore not that D-dimer alone can define breast cancer aggressiveness. Rather, serial D-dimer measurements may contain clinically relevant information when interpreted according to their magnitude, trajectory, persistence, variability, rate of change, treatment responsiveness, and interaction with other biological and clinical variables. A persistently increasing D-dimer concentration may have a different biological meaning from a transient postoperative elevation or a sustained decline during effective anticancer therapy. Artificial intelligence provides a potential analytical framework for distinguishing such complex temporal and contextual patterns (25). Within this proposed framework, D-dimer data could be integrated with tumour stage, molecular subtype, metastatic burden, platelet count, fibrinogen, inflammatory biomarkers, renal and hepatic function, treatment exposure, imaging characteristics, and other relevant variables. Machine-learning models could then investigate whether reproducible multidimensional patterns exist and whether they provide incremental predictive value beyond established prognostic systems. Importantly, this remains a hypothesis-driven research framework. The biological plausibility of integrating coagulation, thromboinflammation, fibrinolysis, and AI does not itself establish clinical validity (26).

### D-dimer as a prognostic biomarker in breast cancer

D-dimer has attracted increasing interest as a potential prognostic biomarker in breast cancer because it reflects the degradation of cross-linked fibrin and, indirectly, the activation of coagulation and fibrinolysis. Several observational studies have reported associations between elevated circulating D-dimer concentrations and advanced tumour stage, greater metastatic burden, cancer-associated thrombosis, disease progression, and reduced survival. Nevertheless, the available evidence remains heterogeneous, and D-dimer should not currently be regarded as an independent or breast cancer-specific prognostic biomarker. Its interpretation requires careful consideration of tumour characteristics, host factors, treatment exposure, comorbidities, and other causes of coagulation activation (27). The prognostic relevance of D-dimer is biologically plausible because aggressive malignancy may generate a sustained procoagulant and thromboinflammatory state. Increasing tumour burden can enhance tissue factor-associated coagulation activation, thrombin generation, fibrin deposition, platelet activation, endothelial perturbation, and subsequent fibrinolysis. Consequently, patients with advanced or biologically aggressive breast cancer may exhibit higher D-dimer concentrations than patients with localized disease. Elevated D-dimer has also been associated in some studies with lymph-node involvement, distant metastasis, treatment resistance, and poorer progression-free or overall survival. However, these associations do not establish that D-dimer itself is a causal determinant of adverse outcomes. Rather, it may function as a downstream indicator of the combined effects of tumour burden, systemic inflammation, haemostatic activation, and coexisting clinical conditions (28).

A major limitation of the existing literature is the substantial variability in the definition of an “elevated” D-dimer. Studies have used different assays, analytical platforms, reporting units, institutional reference intervals, and study-specific thresholds. Some investigations have applied conventional laboratory upper reference limits, whereas others have derived optimal cut-offs retrospectively using receiver operating characteristic analysis. Consequently, a threshold associated with survival or progression in one cohort may not be directly transferable to another. This analytical heterogeneity currently prevents the establishment of a universal D-dimer cut-off for prognostication in breast cancer (29). The reporting of prognostic performance is similarly inconsistent. Although some studies report hazard ratios, odds ratios, receiver operating characteristic curves, or area-under-the-curve estimates, sensitivity, specificity, positive predictive value, negative predictive value, and true-positive and false-positive classifications are not uniformly available. These measures are also strongly dependent on the selected threshold, disease prevalence, clinical setting, outcome definition, and duration of follow-up. Therefore, claims regarding the prognostic accuracy of D-dimer should be based on study-specific data rather than extrapolated across heterogeneous populations. Future studies should report complete diagnostic and prognostic performance metrics and evaluate whether D-dimer provides incremental value beyond established clinicopathological models (30).

Multiple confounders further complicate the prognostic interpretation of D-dimer. Age is particularly relevant because D-dimer concentrations may increase with advancing age independently of malignancy. Infection and systemic inflammation can activate coagulation and fibrinolysis, whilst impaired renal or hepatic function may alter haemostatic balance and biomarker concentrations. Recent surgery, hospitalization, trauma, pregnancy, cardiovascular disease, obesity, immobility, and comorbid inflammatory disorders may also elevate D-dimer. Most importantly, active or occult venous thromboembolism can substantially increase D-dimer concentrations and independently worsen clinical outcomes. Failure to account for these variables may result in confounding and overestimation of the cancer-specific prognostic value of D-dimer (31). Breast cancer-related factors must also be considered. Tumour stage, histological grade, nodal status, metastatic burden, oestrogen receptor status, progesterone receptor status, HER2 expression, triple-negative phenotype, performance status, and treatment modality are established determinants of outcome and may also influence the haemostatic environment. Chemotherapy, surgery, endocrine therapy, targeted therapy, hospitalization, and central venous access may modify thrombotic risk and D-dimer concentrations. Accordingly, prognostic models based on D-dimer should adjust for these variables rather than interpreting D-dimer as an isolated predictor (32). Artificial intelligence could potentially model these nonlinear and time-dependent relationships by integrating serial D-dimer measurements with age, comorbidities, renal and hepatic function, infection status, tumour stage, molecular subtype, metastatic burden, treatment exposure, inflammatory markers, coagulation parameters, and imaging findings. Such an approach may help determine whether distinct D-dimeromic phenotypes provide incremental prognostic information beyond established breast cancer risk models. However, this proposition remains hypothesis-generating, as AI-enabled D-dimeromics has not yet been prospectively validated as a clinical prognostic system in breast cancer. Therefore, the current evidence supports a cautious interpretation. D-dimer is a biologically plausible and potentially informative marker of the systemic haemostatic and thromboinflammatory consequences of breast cancer, but it is nonspecific and highly context dependent. Its future prognostic value may lie less in an isolated cut-off and more in standardized, longitudinal, multimodal assessment. Prospective multicentre studies should harmonize D-dimer assays and units, prespecify thresholds, report sensitivity, specificity, positive and negative predictive values where appropriate, adjust for major confounders, include negative findings, and evaluate incremental predictive value against established clinicopathological models. Such evidence will be essential before D-dimer, or the broader D-dimeromics framework, can be incorporated into routine prognostic decision-making in breast oncology (**Table 1**).

**Table 1:** D-dimer as a prognostic biomarker in breast cancer.

Clinical domain	D-dimer-related observation	Biological interpretation	Prognostic implication	Clinical relevance
Tumour burden	Elevated D-dimer correlates with larger tumour size and advanced TNM stage	Increased tissue factor expression, fibrin formation, and tumour-driven coagulation activation	Indicates high tumour load and aggressive disease biology	Supports initial risk stratification at diagnosis
Lymph node involvement	Higher D-dimer levels observed in node-positive disease	Enhanced metastatic dissemination and lymphovascular invasion	Predicts regional spread and worse outcomes	Helps refine staging and treatment intensity
Distant metastasis	Significantly elevated D-dimer in patients with bone, liver, lung, or brain metastases	Fibrin scaffolding facilitates circulating tumour cell survival and colonization	Strong indicator of systemic disease dissemination	May signal occult metastatic disease
Molecular subtype	Higher levels in triple-negative and HER2-enriched breast cancer	Aggressive tumour biology with increased inflammation and coagulation activation	Reflects intrinsically high-risk tumour phenotypes	Useful adjunct to molecular classification
Inflammatory state	Positive correlation with CRP, IL-6, and NLR	Thromb oinflammation driven by cytokine-mediated coagulation activation	Indicates systemic inflammatory burden	Enhances prognostic accuracy when combined with inflammatory markers
Treatment response	Persistently elevated or rising D-dimer during therapy	Ongoing tumour activity, resistance, or incomplete response	Early indicator of treatment failure	Supports early therapy modification
Disease recurrence	Increased D-dimer precedes clinical or radiological recurrence	Minimal residual disease and microthrombotic activity	Predicts early relapse	Useful for surveillance strategies
Overall survival	High baseline D-dimer associated with reduced OS	Reflects cumulative tumour aggressiveness and systemic involvement	Strong predictor of poor survival outcomes	Helps identify ultra-high-risk patients
Progression-free survival	Elevated D-dimer associated with shorter PFS	Ongoing tumour progression and biological instability	Indicates rapid disease evolution	Guides treatment escalation decisions
Post-treatment monitoring	Dynamic changes in D-dimer correlate with disease trajectory	Real-time reflection of tumour-host interactions	Enables longitudinal risk assessment	Useful for follow-up and monitoring

### Artificial intelligence as the engine of D-dimeromics

Artificial intelligence (AI) provides the computational foundation for the proposed concept of D-dimeromics because the prognostic meaning of D-dimer in breast cancer is unlikely to be captured adequately by a single measurement or universal threshold. D-dimer concentrations are dynamic, nonspecific, and influenced by tumour burden, treatment, thrombosis, inflammation, age, comorbidities, renal and hepatic function, infection, surgery, and other clinical conditions. The central hypothesis of AI-enabled D-dimeromics is therefore that clinically relevant information may reside within complex interactions amongst longitudinal D-dimer trajectories and multimodal patient data. AI could potentially identify these nonlinear, time-dependent patterns and determine whether they improve risk prediction beyond established clinicopathological models. However, no prospectively validated AI-driven D-dimeromics system currently exists for routine breast cancer care; consequently, the framework remains hypothesis-generating (33). The development of a clinically meaningful D-dimeromics model would require clearly defined and harmonized data domains. Clinical data should include age, body mass index, performance status, comorbidities, previous venous thromboembolism (VTE), infection, hospitalization, immobility, recent surgery, cardiovascular disease, medication exposure, and other conditions capable of influencing D-dimer. Breast cancer-specific variables should include tumour stage, histological type and grade, tumour size, nodal involvement, metastatic sites and burden, and disease status. Treatment variables should capture surgery, chemotherapy, endocrine therapy, radiotherapy, HER2-directed treatment, immunotherapy, central venous access, and the timing of each intervention relative to biomarker measurement. Without such contextual information, an algorithm may incorrectly attribute non-malignant or treatment-related D-dimer elevations to tumour aggressiveness (34).

Laboratory data would constitute the core biological layer of the model. Beyond serial D-dimer measurements, relevant variables may include platelet count, fibrinogen, prothrombin time, activated partial thromboplastin time, full blood count-derived inflammatory indices, C-reactive protein, renal function, hepatic function, and other validated haemostatic or inflammatory markers. Where available, more specialized markers of thrombin generation, endothelial activation, platelet activation, or inflammation could be incorporated. Importantly, the D-dimer input should not be limited to a binary normal/elevated classification. Potential computational features include baseline concentration, maximum value, absolute and percentage change, rate of change, persistence of elevation, intra-patient variability, time-weighted average, and area under the longitudinal concentration curve. These features could provide a more comprehensive representation of the patient’s evolving thrombo-fibrinolytic phenotype (35). Pathological and molecular data may further contextualize D-dimer dynamics. Variables such as oestrogen receptor, progesterone receptor and HER2 status, Ki-67, histological grade, and molecular subtype could allow algorithms to investigate whether D-dimer trajectories differ across biologically distinct forms of breast cancer. Genomic, transcriptomic, proteomic, circulating tumour DNA, or circulating tumour cell data could potentially be incorporated in research settings. Such integration could help determine whether systemic thromboinflammatory signals provide complementary information to tumour-specific molecular biomarkers rather than merely duplicating established measures of disease burden (36).

Imaging data represent another potentially important component. Conventional radiological variables such as primary tumour dimensions, nodal involvement, number and location of metastatic lesions, and longitudinal changes in tumour burden could be integrated with laboratory trajectories. More advanced models might incorporate quantitative radiomic features extracted from mammography, ultrasound, computed tomography, magnetic resonance imaging, or positron emission tomography. The objective would be to examine whether changes in D-dimer and other systemic biomarkers correspond to spatial and temporal changes in tumour phenotype. However, imaging heterogeneity, differences in acquisition protocols, segmentation variability, and the computational demands of multimodal integration remain substantial challenges (37). The choice of AI method should be determined by the clinical question and data structure rather than algorithmic novelty. Penalized regression approaches, including least absolute shrinkage and selection operator methods, may provide relatively interpretable models and perform feature selection in datasets containing numerous correlated variables. However, they may inadequately capture highly nonlinear interactions unless these are explicitly modelled. Support vector machines (SVMs) can perform well in high-dimensional datasets and may identify complex decision boundaries, but their performance depends on kernel and hyperparameter selection, whilst clinical interpretation can be difficult (38).

Tree-based ensemble methods, including random forests and gradient-boosting algorithms, may be particularly useful for integrating heterogeneous clinical and laboratory variables because they can model nonlinear relationships and interactions without requiring strict distributional assumptions. Nevertheless, they remain vulnerable to overfitting, class imbalance, dataset shift, and reduced transportability across institutions. Apparent high performance in a single retrospective dataset may therefore fail during external validation. Longitudinal D-dimeromics may require models capable of analysing temporal sequences. Recurrent neural networks (RNNs) and long short-term memory architectures can theoretically learn patterns from serial D-dimer and clinical measurements. However, healthcare data are often characterized by irregular sampling intervals, missing observations, changing treatment exposures, and informative testing frequency. For example, clinically deteriorating patients may undergo D-dimer testing more frequently than stable patients, creating measurement bias that an algorithm could mistakenly interpret as biological risk. Temporal models must therefore account for both the biomarker trajectory and the clinical process generating the data (39).

Deep-learning approaches may enable integration of large-scale imaging, molecular, and longitudinal datasets, but they require substantial sample sizes and computational resources. Their complexity may also reduce interpretability and increase the risk of overfitting, particularly in relatively small breast cancer cohorts. Consequently, a complex deep-learning architecture should not automatically be preferred over a simpler and more transparent model. In early D-dimeromics research, parsimonious models based on routinely available clinical and laboratory variables may provide greater reproducibility and clinical feasibility. Unsupervised learning offers another potential approach. Clustering and latent-class methods could be used to investigate whether reproducible D-dimeromic phenotypes exist without imposing predefined risk categories. For example, patients might theoretically cluster into stable-low, treatment-responsive, persistently elevated, rapidly increasing, or thromboinflammatory D-dimer trajectories. However, data-driven clusters are not inherently clinically meaningful. Any identified phenotype would require replication in independent populations and demonstration of associations with prespecified outcomes such as VTE, progression, recurrence, or mortality (38).

The outcomes used to train AI-enabled D-dimeromics models must also be clearly defined. A single model should not indiscriminately combine biologically distinct endpoints. Prediction of VTE, early recurrence, treatment resistance, progression-free survival, and overall survival may require different predictors, time horizons, modelling strategies, and validation procedures. For survival outcomes, time-to-event and competing-risk methods may be more appropriate than simple binary classification. The intended clinical use of each model should therefore be specified before model development. Data quality represents a fundamental challenge. D-dimer measurements differ across assays, analytical platforms, units, reference ranges, and institutions. Missing data, inconsistent sampling schedules, retrospective selection bias, and incomplete documentation of confounders may substantially distort model development. Harmonization of measurement units and timing is therefore essential. Multicentre datasets should also account for differences in patient demographics, healthcare systems, treatment protocols, and laboratory practices to reduce algorithmic bias and improve generalizability (39). Model performance should not be evaluated solely by discrimination metrics such as accuracy or the area under the receiver operating characteristic curve. Calibration is essential because predicted probabilities must correspond reasonably to observed clinical risks. Sensitivity, specificity, positive and negative predictive values, precision–recall measures, and clinically appropriate time-dependent metrics should be reported where relevant. Decision-curve analysis may help determine whether the model provides net clinical benefit compared with existing strategies. Crucially, an AI model should demonstrate incremental predictive value beyond established clinicopathological prognostic models, rather than merely showing that D-dimer is associated with adverse outcomes.

**Figure 2:**
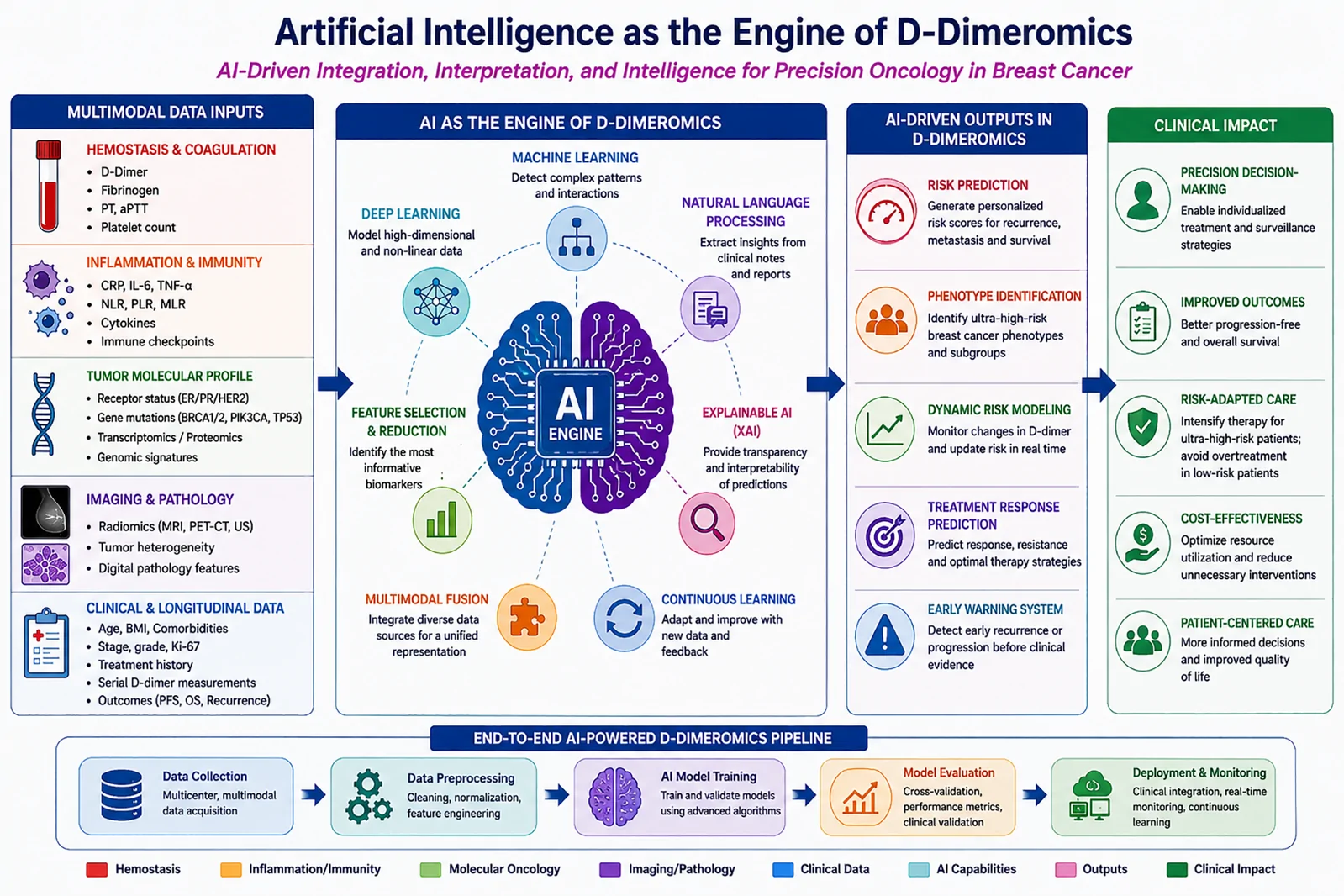
Artificial intelligence as the engine of D-dimeromics.

Explainable AI is particularly important for D-dimeromics because D-dimer is inherently nonspecific. Methods such as feature-importance analysis and SHapley Additive exPlanations may help identify the variables contributing to an individual prediction. For example, a high-risk output might be driven by persistent D-dimer elevation combined with increasing metastatic burden and inflammatory markers rather than by D-dimer alone. Nevertheless, computational explainability does not establish biological causality, and explanations generated by an algorithm should be interpreted cautiously. A proposed AI-enabled D-dimeromics workflow would therefore involve multimodal data acquisition → assay and temporal harmonization → D-dimer trajectory extraction → integration of clinical, laboratory, pathological, molecular, and imaging variables → model development → explainability analysis → external validation → prospective clinical evaluation. This framework should be compared directly with standard clinical prediction approaches to determine whether its additional complexity produces meaningful improvements in patient care (**Figure 2**).

### AI-enabled D-dimeromics for identifying ultra-high-risk breast cancer phenotypes

The identification of patients at exceptionally high risk of rapid progression, treatment failure, cancer-associated thrombosis, early recurrence, or mortality remains an important challenge in breast oncology. Conventional risk stratification relies on tumour stage, histological grade, nodal status, molecular subtype, genomic signatures, metastatic burden, and treatment response. Although these variables provide substantial prognostic information, they may not fully capture the dynamic systemic consequences of aggressive cancer, particularly the evolving interactions amongst tumour burden, coagulation activation, fibrinolysis, inflammation, endothelial dysfunction, and treatment exposure. AI-enabled D-dimeromics is proposed as a hypothesis-driven framework for investigating whether longitudinal D-dimer patterns, interpreted within this broader biological and clinical context, can identify patients whose risk exceeds that captured by established prognostic models (39). A critical distinction must be made between high-risk and ultra-high-risk breast cancer. High-risk disease is already defined in different clinical contexts using established clinicopathological and molecular characteristics, although definitions vary according to disease setting and outcome of interest. In contrast, ultra-high-risk breast cancer is not currently a universally recognized or standardized clinical category. Within the proposed D-dimeromics framework, the term is used as an operational research construct to describe patients with an exceptionally high, objectively quantified probability of a prespecified adverse outcome within a defined time horizon despite conventional risk assessment. Such outcomes may include early disease progression, early recurrence, treatment resistance, cancer-associated venous thromboembolism (VTE), or mortality (40).

Accordingly, ultra-high-risk status should not be assigned solely on the basis of an elevated D-dimer concentration. It should ultimately be defined using prospectively prespecified outcome thresholds, validated prediction models, appropriate time horizons, and demonstration of incremental risk beyond established prognostic systems. For example, a future model might classify a patient as ultra-high risk only when the predicted probability of a clearly defined endpoint exceeds a validated threshold associated with clinically meaningful consequences. The precise threshold would need to be derived and externally validated rather than arbitrarily selected. This distinction is essential to prevent the proposed terminology from being interpreted as an established clinical classification (41). The central hypothesis is that ultra-high-risk disease may be characterized by multidimensional biological convergence rather than by a single adverse variable. A patient with persistent or rapidly increasing D-dimer, increasing metastatic burden, systemic inflammation, adverse tumour biology, and poor treatment response may represent a fundamentally different risk state from a patient with an isolated transient D-dimer elevation. AI could potentially identify these complex interactions and determine whether they define reproducible phenotypes associated with extreme outcome risks.

The D-dimer domain could include baseline concentration, peak concentration, persistence of elevation, velocity of change, intra-patient variability, and response to treatment. A single elevated value may be nonspecific, whereas persistent or accelerating changes could potentially provide different information when interpreted alongside other clinical variables. The tumour domain should incorporate stage, histological grade, nodal status, molecular subtype, proliferation indices, metastatic sites, and quantitative disease burden. Where available, genomic, transcriptomic, circulating tumour DNA, or other molecular information could further characterize tumour aggressiveness. The host and thromboinflammatory domain may include age, comorbidities, platelet count, fibrinogen, inflammatory biomarkers, renal and hepatic function, previous VTE, infection, performance status, and other factors capable of modifying D-dimer concentrations or clinical outcomes. The treatment-response domain should capture the type and timing of therapy, radiological response, biomarker changes, treatment interruptions, and disease progression. Because risk is dynamic, a patient’s classification may change during the disease course (41).

Rather than forcing all patients into a binary low-risk/high-risk classification, AI could investigate whether distinct longitudinal phenotypes exist. A stable-low D-dimer phenotype might be characterized by consistently low or minimally changing D-dimer concentrations without adverse multimodal signals. A treatment-responsive phenotype could exhibit initially elevated D-dimer followed by sustained reduction alongside objective tumour response. A persistent thromboinflammatory phenotype might demonstrate sustained D-dimer elevation accompanied by inflammatory and haemostatic abnormalities. A rapidly evolving phenotype could be characterized by accelerating D-dimer trajectories, increasing tumour burden, and deteriorating clinical indicators. A potential ultra-high-risk multimodal phenotype would represent the convergence of several adverse signals rather than an isolated laboratory abnormality. Hypothetically, this could include persistent or rapidly rising D-dimer, increasing metastatic burden, adverse molecular characteristics, elevated inflammatory markers, and inadequate treatment response. Whether such a phenotype truly exists, is reproducible across populations, and predicts outcomes independently of established variables remains to be determined prospectively (42).

An important methodological consideration is that “ultra-high risk” should be outcome specific. A patient at exceptionally high risk of VTE is not necessarily the same as a patient at exceptionally high risk of early cancer recurrence. Consequently, separate models may be required for distinct clinical endpoints. For VTE prediction, relevant variables may include serial D-dimer, previous thrombosis, platelet count, body mass index, immobility, hospitalization, central venous access, systemic therapy, metastatic disease, and comorbidities. For progression or recurrence, the model may place greater emphasis on D-dimer trajectories, tumour burden, molecular subtype, treatment response, imaging findings, and tumour-specific biomarkers. For mortality prediction, performance status, metastatic burden, organ function, systemic inflammation, treatment response, and competing causes of death may become particularly important. This endpoint-specific approach avoids the development of a single opaque “risk score” that combines biologically and clinically distinct outcomes (43). Traditional prognostic classifications are often based on variables measured at diagnosis. However, the biological state of a patient with breast cancer changes during treatment and disease progression. AI-enabled D-dimeromics therefore proposes a dynamic risk-state model in which risk estimates are updated as new information becomes available. Such a model could theoretically distinguish transient D-dimer elevations related to surgery or acute illness from persistent patterns occurring alongside progressive disease. However, this requires accurate time-stamped data and careful modelling of treatment exposures and competing clinical events.

The purpose of identifying an ultra-high-risk phenotype would not be simply to generate a higher numerical risk score. A clinically meaningful classification must ultimately support an evidence-based action. Following appropriate validation, an AI-generated high-risk signal could potentially prompt more detailed clinical assessment, evaluation for alternative causes of D-dimer elevation, closer surveillance, or multidisciplinary review. It could also support clinical-trial enrichment by identifying patients with particularly adverse biological trajectories. One of the greatest challenges is distinguishing tumour-associated D-dimer elevation from competing causes. Age, infection, renal or hepatic dysfunction, recent surgery, hospitalization, trauma, pregnancy, cardiovascular disease, inflammation, and established VTE may all increase D-dimer concentrations. Some of these factors also independently predict poor survival, creating substantial potential for confounding. An AI model that predicts poor outcomes from elevated D-dimer without appropriately accounting for these factors may simply reproduce known associations between comorbidity and mortality. Therefore, robust D-dimeromic models must incorporate relevant confounders and evaluate whether D-dimer-related features provide independent and incremental prognostic information beyond established predictors (44, 45).

The proposed phenotype requires a rigorous validation pathway. Initial model development should use clearly defined patient populations, standardized D-dimer measurements, prespecified outcomes, and appropriate handling of missing and longitudinal data. Internal validation should be followed by temporal and external validation across independent institutions and populations. Model evaluation should include discrimination, calibration, sensitivity, specificity, positive and negative predictive values where appropriate, and decision-curve analysis. Comparisons with established clinicopathological models are essential. A D-dimeromics model should not be considered clinically valuable merely because it predicts an outcome; it must demonstrate that it improves prediction or decision-making beyond existing approaches. The term “ultra-high risk” should also be linked to validated absolute-risk thresholds rather than relative-risk estimates alone. Thresholds should be selected according to the intended clinical use and the consequences of false-positive and false-negative classifications. At present, there is no prospectively validated AI-enabled D-dimeromics model that identifies an established ultra-high-risk breast cancer phenotype for routine clinical practice. The proposed framework therefore represents a research hypothesis rather than a validated classification system. Existing evidence supporting associations amongst D-dimer, coagulation activation, thrombosis, tumour progression, and adverse outcomes provides biological plausibility, but it does not establish the clinical validity of the integrated D-dimeromics concept (46, 47) (**Table 2**).

**Table 2:** AI-enabled D-dimeromics for identifying ultra-high-risk breast cancer phenotypes.

AI component	Data inputs	Analytical function	Output in D-dimeromics	Role in detecting ultra-high-risk phenotypes	Clinical utility
Random Forest	D-dimer, fibrinogen, CRP, NLR, tumour stage, receptor status	Nonlinear feature selection and classification	Ranked importance of D-dimer within risk hierarchy	Identifies key drivers of thromboinflammatory risk profiles	Early risk stratification and variable prioritization
Gradient Boosting Machines (XGBoost/LightGBM)	Multimodal clinical + laboratory + pathology data	High-accuracy predictive modelling of outcomes	Composite risk score for recurrence and metastasis	Detects subtle interactions between D-dimer and tumour biology	Prognostic scoring and survival prediction
Support Vector Machines (SVM)	Standardized biomarker panels and molecular subtype data	Binary classification of high vs low-risk disease	Decision boundary separating ultra-high-risk phenotypes	Enhances discrimination of aggressive tumour subsets	Diagnostic support tool in early classification
Artificial Neural Networks (ANN)	Large-scale integrated clinical and biomarker datasets	Pattern recognition in nonlinear biological systems	Deep risk probability scores	Captures complex thromboinflammatory–tumour interactions	Advanced risk modelling in heterogeneous datasets
Convolutional Neural Networks (CNN)	Mammography, MRI, PET-CT radiomic features	Image feature extraction and spatial pattern recognition	Imaging-derived aggressiveness score	Links radiological heterogeneity with D-dimer elevation	Imaging-based phenotyping and staging refinement
Recurrent Neural Networks (RNN)/LSTM	Serial D-dimer measurements and longitudinal clinical data	Temporal sequence modelling	Trajectory-based risk evolution curves	Detects rising or unstable D-dimer patterns indicating progression	Early warning of relapse or treatment failure
Transformer Models	Multimodal time-series (omics, imaging, lab data)	Context-aware integration of long-range dependencies	Unified multimodal risk embedding	Identifies hidden temporal and cross-domain interactions	High-level predictive clinical decision support
Clustering Algorithms (K-means, hierarchical clustering)	Integrated biomarker + imaging + genomic datasets	Unsupervised phenotype discovery	Identification of latent patient subgroups	Reveals ultra-high-risk biological clusters	Patient stratification for personalized therapy
SHAP (Explainable AI layer)	Outputs from predictive models	Feature attribution and interpretability	Contribution score of D-dimer and other variables	Clarifies role of D-dimer in risk classification	Enhances clinician interpretability and trust
LIME (Explainability tool)	Individual patient-level model outputs	Local explanation of predictions	Patient-specific risk rationale	Identifies why a patient is classified as ultra-high-risk	Supports individualized clinical decision-making

To further illustrate the potential clinical utility of AI-enabled D-dimeromics, the proposed framework envisions the generation of individualized probabilistic risk estimates rather than reliance on fixed biomarker cut-off values. Following appropriate model development and rigorous external validation, AI algorithms could integrate longitudinal D-dimer measurements with clinicopathological variables, imaging features, genomic alterations, inflammatory biomarkers, and treatment-related factors to estimate patient-specific risks for clinically meaningful outcomes. For example, the model could provide individualized probabilities for events such as 2-year overall mortality, 6-month disease recurrence, development of distant metastasis, cancer-associated venous thromboembolism, or progression despite systemic therapy. These outputs would enable dynamic risk stratification and support personalized surveillance, therapeutic selection, and multidisciplinary clinical decision-making. These examples are presented solely as conceptual illustrations of the intended functionality of AI-enabled D-dimeromics and should not be interpreted as validated clinical thresholds or decision-making criteria. At present, no standardized or universally accepted AI-derived probability thresholds exist for defining an ultra-high-risk phenotype based on D-dimer-integrated models. Establishing such thresholds will require large prospective multicentre studies, harmonized D-dimer assay methodologies, standardized outcome definitions, and robust external validation across diverse patient populations. Until these prerequisites are met, the proposed ultra-high-risk phenotype should be regarded as an operational research construct intended to guide future biomarker discovery and predictive model development rather than an immediately applicable clinical classification.

### Clinical implications and practical application pathways of the ultra-high-risk breast cancer phenotype

The concept of an ultra-high-risk breast cancer phenotype proposed in this review represents a hypothesis-driven precision oncology framework designed to identify patients with a markedly increased probability of adverse clinical outcomes through the integration of longitudinal D-dimer dynamics, multimodal biomarkers, and artificial intelligence. Rather than relying solely on conventional clinicopathological variables or static molecular classifications, this framework seeks to capture the dynamic biological evolution of disease by incorporating indicators of tumour-associated hypercoagulability, systemic inflammation, immune dysregulation, angiogenesis, and metastatic potential. At present, this phenotype should be regarded as a conceptual model requiring prospective validation before clinical implementation (39). One of the principal clinical implications of this framework is more refined prognostic stratification. Current risk assessment in breast cancer primarily depends on tumour size, nodal status, histological grade, receptor status, molecular subtype, proliferation indices, and selected genomic assays. Although these parameters provide valuable prognostic information, they may not fully reflect the evolving biological processes that drive recurrence, metastatic dissemination, treatment resistance, or cancer-associated thrombosis. AI-enabled D-dimeromics has the potential to complement existing prognostic models by integrating longitudinal D-dimer trajectories with radiomic, pathomic, genomic, liquid biopsy, and clinical data to generate continuously updated individualized risk profiles (40).

The proposed framework could also facilitate earlier identification of patients at risk of metastatic progression. Persistent or progressively increasing D-dimer concentrations, particularly when interpreted alongside circulating tumour DNA (ctDNA), circulating tumour cells, advanced imaging features, and molecular biomarkers, may reflect active tumour dissemination before conventional clinical or radiological evidence becomes apparent. AI algorithms capable of recognizing complex temporal relationships amongst these variables could theoretically identify patients requiring intensified surveillance and earlier diagnostic evaluation. However, this application remains investigational and requires validation in prospective longitudinal studies (41). Another important potential application involves personalized treatment stratification. AI-enabled D-dimeromics may assist multidisciplinary oncology teams in identifying patients who are more likely to benefit from intensified systemic therapy, enrolment in biomarker-driven clinical trials, or closer therapeutic monitoring. For example, patients predicted to have biologically aggressive disease characterized by persistent hypercoagulability, inflammatory activation, and evolving molecular resistance may warrant more frequent clinical assessment, serial biomarker monitoring, or adaptive treatment strategies. Importantly, such decisions should continue to rely on established clinical guidelines until the proposed framework has undergone rigorous clinical validation (42).

The framework may also contribute to individualized thrombosis risk assessment. Cancer-associated thrombosis remains a major cause of morbidity and mortality in patients with breast cancer, yet existing risk prediction models demonstrate only moderate predictive performance. Integrating longitudinal D-dimer kinetics with patient characteristics, treatment variables, inflammatory biomarkers, platelet indices, and AI-derived predictive models may improve identification of individuals at particularly high thrombotic risk. Following prospective validation, this approach could potentially support more individualized thromboprophylaxis strategies whilst balancing thrombotic and bleeding risks (43). AI-enabled D-dimeromics also offers opportunities for dynamic treatment monitoring. Rather than relying exclusively on periodic imaging, continuously updated AI models incorporating serial D-dimer measurements, liquid biopsy results, radiomic changes, and clinical parameters could provide early indications of therapeutic response, emerging resistance, minimal residual disease, or impending recurrence. Such dynamic monitoring may eventually facilitate more timely treatment modifications and improve long-term disease control. Nevertheless, this concept remains exploratory and should currently be considered a future research direction (44).

An additional application involves precision surveillance after primary treatment. Patients identified by AI-enabled D-dimeromics as belonging to an ultra-high-risk phenotype could potentially undergo individualized follow-up schedules that include more frequent biomarker assessment, targeted imaging, and clinical review compared with standard surveillance protocols. Conversely, patients classified as lower risk might avoid unnecessary investigations, thereby improving resource utilization and reducing patient burden. Prospective studies will be necessary to determine whether risk-adapted surveillance based on AI-enabled D-dimeromics improves clinical outcomes and healthcare efficiency (45). The proposed framework may also support patient selection for precision oncology clinical trials. By integrating biological, molecular, imaging, and coagulation signatures, AI-enabled D-dimeromics could facilitate identification of patients with highly aggressive disease who may derive greater benefit from investigational therapies targeting angiogenesis, immunomodulation, coagulation pathways, or metastatic progression. This approach may enhance trial enrichment strategies and improve the evaluation of novel therapeutic interventions (46). From a health systems perspective, implementation would require a stepwise translational pathway. The initial phase should focus on multicentre observational studies to validate the association between integrated D-dimeromic signatures and clinically meaningful outcomes across diverse patient populations. Subsequent studies should externally validate AI prediction models using standardized laboratory assays, harmonized imaging protocols, and interoperable clinical datasets. Following successful validation, prospective implementation studies should evaluate the incremental prognostic value, clinical utility, cost-effectiveness, and workflow integration of AI-enabled D-dimeromics compared with existing risk assessment tools. Only after demonstrating robust analytical validity, clinical validity, and clinical utility should the framework be considered for incorporation into routine decision-support systems and clinical practice guidelines (47).

### Integration of AI-enabled D-dimeromics with precision oncology

Precision oncology aims to tailor cancer prevention, diagnosis, treatment, and surveillance to the biological and clinical characteristics of the individual patient. In breast cancer, this approach has traditionally focused on tumour-centred variables, including histological characteristics, oestrogen receptor (ER), progesterone receptor (PR), and human epidermal growth factor receptor 2 (HER2) status, genomic signatures, and actionable molecular alterations. Although these factors have transformed treatment selection, they do not fully capture the dynamic systemic consequences of malignancy. AI-enabled D-dimeromics is proposed as a complementary, host–tumour interface framework that integrates longitudinal D-dimer patterns with clinical, pathological, molecular, inflammatory, haemostatic, treatment, and imaging data. Its potential contribution to precision oncology lies not in replacing established biomarkers, but in determining whether dynamic thrombo-fibrinolytic information can provide additional, clinically actionable risk information (29). A central principle of this integration is that D-dimer should not be interpreted in isolation. Elevated D-dimer is nonspecific and may reflect cancer-associated coagulation activation, venous thromboembolism (VTE), infection, inflammation, surgery, hospitalization, renal or hepatic dysfunction, cardiovascular disease, or other conditions. Therefore, a precision D-dimeromics model would contextualize D-dimer magnitude and trajectory against the patient’s tumour characteristics, comorbidities, treatment exposures, organ function, inflammatory status, and disease course. AI could potentially identify combinations of variables that distinguish transient or explainable D-dimer elevations from persistent multimodal patterns associated with adverse outcomes. This remains a hypothesis requiring prospective validation (48, 49).

At diagnosis, oncologists routinely integrate tumour stage, grade, nodal status, molecular subtype, performance status, and, where appropriate, genomic assays to estimate prognosis and guide treatment. AI-enabled D-dimeromics could potentially add a systemic biological dimension to this assessment. For example, a model might investigate whether persistent haemostatic activation combined with high inflammatory burden and adverse tumour characteristics identifies patients whose observed risk exceeds that predicted by conventional clinicopathological models. The clinically relevant question is therefore not whether an elevated D-dimer independently defines aggressive breast cancer, but whether incorporating standardized D-dimer-related features produces measurable incremental predictive value. Any proposed model should be compared directly with established prognostic approaches using discrimination, calibration, reclassification, and decision-curve analyses (50, 51). One potential application of D-dimeromics is longitudinal risk assessment during anticancer therapy. Conventional assessments often occur at predefined clinical or imaging intervals, whereas laboratory data may provide more frequent information about changes in the patient’s systemic state. Serial D-dimer measurements could theoretically be analysed alongside tumour markers, inflammatory indices, organ function, symptoms, and imaging response. For example, declining D-dimer concentrations occurring alongside objective tumour regression may represent a different biological trajectory from persistently increasing D-dimer accompanied by increasing tumour burden and systemic inflammation. Conversely, an abrupt increase in D-dimer following surgery, during infection, or in association with suspected VTE may have an entirely different clinical interpretation. AI could potentially integrate these temporal relationships rather than assigning identical meaning to numerically similar D-dimer values. However, a worsening D-dimeromic risk signal should not itself be considered evidence of cancer progression or treatment failure. Its potential role would be to support clinical reassessment when interpreted alongside symptoms, examination findings, imaging, and other established investigations (52, 53).

VTE is an important complication of malignancy and anticancer treatment. Risk varies considerably amongst patients according to tumour burden, previous thrombosis, treatment, hospitalization, immobility, central venous access, comorbidities, and other factors. A dynamic D-dimeromics model could potentially integrate serial D-dimer measurements with these variables to generate individualized, time-updated estimates of thrombotic risk. For the practicing oncologist, such a validated model could theoretically identify patients who warrant heightened clinical vigilance or further assessment for thromboembolic disease. Importantly, an AI-generated risk estimate should not independently determine anticoagulant therapy. Decisions concerning diagnostic imaging, prophylaxis, or treatment must remain based on validated clinical pathways and an individualized assessment of thrombotic and bleeding risks (54). A potentially important precision-oncology application is the identification of discordance between conventional tumour assessment and the patient’s evolving systemic biological state. A patient may demonstrate apparently stable disease on one assessment whilst developing persistent changes in coagulation, inflammation, organ function, or other biomarkers. Conversely, isolated D-dimer elevation may occur despite stable cancer because of a non-malignant condition. AI-enabled multimodal analysis could potentially identify such discordance and quantify whether it is associated with subsequent clinical events. This could provide oncologists with a hypothesis-generating alert for closer assessment rather than an autonomous diagnostic conclusion. The clinical value of such alerts would require prospective demonstration that they improve outcomes without causing excessive investigations, false-positive findings, or unnecessary treatment (55).

D-dimeromics may also complement tumour-specific precision biomarkers. Genomic profiling characterizes molecular alterations, whilst circulating tumour DNA may provide information on molecular residual disease and evolving tumour burden. Imaging provides anatomical and functional information. D-dimer, by contrast, may reflect downstream systemic consequences of tumour–host interactions. Following curative-intent treatment, surveillance strategies are generally guided by established clinical recommendations rather than experimental biomarkers. AI-enabled D-dimeromics could, in future research, investigate whether dynamic multimodal risk estimates identify subgroups with meaningfully different probabilities of recurrence or complications. If prospectively validated, such models might eventually contribute to risk-adapted follow-up by helping determine which patients could benefit from closer clinical assessment. However, there is currently insufficient evidence to recommend D-dimer-guided surveillance, and unnecessary testing may increase false-positive findings, anxiety, healthcare costs, and potentially avoidable interventions. Clinical utility must therefore be demonstrated before implementation (56). The most appropriate future role of AI-enabled D-dimeromics would be as a clinical decision-support system, not an autonomous decision-maker. A clinically useful output should provide an interpretable estimate of risk and identify the variables contributing to that estimate. For example, an oncologist might receive a time-updated risk estimate indicating that the major contributing factors are a persistent D-dimer increase, increasing metastatic burden, inflammatory activation, and declining performance status. Such information could be reviewed within multidisciplinary care involving oncology, haematology, radiology, pathology, and other relevant specialties. Explainability is essential because clinicians must distinguish plausible biological signals from confounding, measurement error, and algorithmic artefacts (**Table 3**) (63).

**Table 3:** Summary of key clinical studies evaluating D-dimer in breast cancer prognosis.

Study (year)	Study design	Study population (n)	Disease setting	Key findings related to D-dimer	Diagnostic/prognostic performance	Major limitations	Clinical implication
(57)	Prospective observational	104	Newly diagnosed breast cancer	Elevated D-dimer associated with advanced clinical stage and metastatic disease	Independent predictor of advanced disease	Small cohort; single centre	D-dimer may reflect tumour burden
(22)	Prospective cohort	122	Metastatic breast cancer	Increased D-dimer correlated with tumour progression and shorter survival	Significant prognostic biomarker for survival	Limited molecular characterization	Supports D-dimer as a marker of aggressive disease
(58)	Prospective study	189	Operable and metastatic breast cancer	Higher D-dimer observed in metastatic patients than localized disease	Associated with reduced overall survival	Moderate sample size	Useful prognostic indicator during staging
(27)	Case-control	160	Breast cancer vs healthy controls	D-dimer significantly elevated in breast cancer patients	Good discrimination between cases and controls	Cross-sectional design	Suggests value as an adjunct diagnostic biomarker
(59)	Prospective multicentre cohort	819 cancer patients (breast cancer subgroup included)	Mixed solid tumours	Elevated D-dimer predicted venous thromboembolism and mortality	Independent predictor after multivariable adjustment	Breast cancer subgroup not analysed separately	Demonstrates utility in thrombotic risk assessment
(60)	Retrospective cohort	247	Breast cancer surgery	Preoperative D-dimer associated with lymph node metastasis and recurrence	Significant predictor of recurrence	Retrospective design	Potential preoperative prognostic biomarker
(21)	Retrospective cohort	356	Invasive breast carcinoma	Elevated D-dimer independently predicted poorer disease-free and overall survival	Independent prognostic factor in Cox regression	Single institution	May improve risk stratification
(61)	Prospective observational	420	Breast cancer receiving chemotherapy	Persistently elevated D-dimer associated with treatment resistance and disease progression	Dynamic monitoring improved prognostic assessment	Limited follow-up duration	Serial D-dimer measurements may monitor treatment response
(61)	Prospective validation	Mixed cancer cohort	Solid malignancies	D-dimer improved prediction of cancer-associated thrombosis when combined with clinical variables	Improved risk discrimination over clinical models alone	Not breast cancer-specific	Supports multimarker predictive modelling
Recent AI-based Biomarker Studies (2023–2025) (62)	AI/ML observational studies	Variable	Precision oncology	Machine learning integrating coagulation biomarkers with clinical and imaging data improved prognostic prediction compared with single biomarkers	Higher AUCs than conventional regression models	Limited external validation; heterogeneous datasets	Supports development of AI-enabled D-dimeromics

AI, Artificial Intelligence; AUC, Area Under the Receiver Operating Characteristic Curve; Cox, Cox proportional hazards regression; HR, Hazard Ratio; ML, Machine Learning; TNM, Tumour-Node-Metastasis; VTE, Venous Thromboembolism.

**Figure 3:**
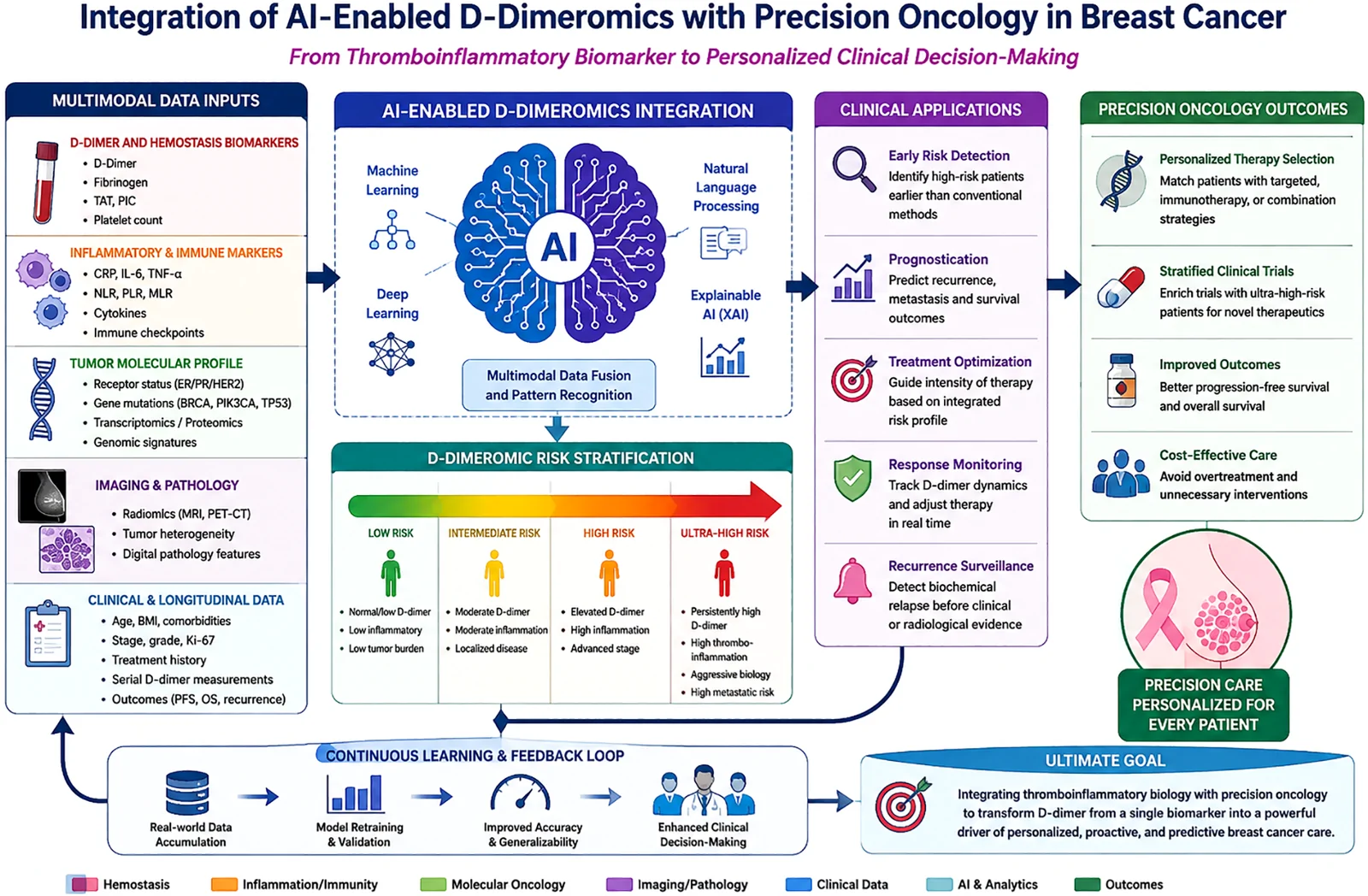
Integration with precision oncology.

D-dimeromics may initially have greater value in research than in routine clinical care. AI-derived phenotypes could potentially enrich clinical trials by identifying patients with specific thromboinflammatory or extreme-risk profiles. This may facilitate investigation of whether particular biological subgroups respond differently to therapeutic or supportive-care strategies. The framework could also support translational research into relationships amongst tumour biology, coagulation, inflammation, thrombosis, and treatment response. Importantly, data-driven phenotypes should not be assumed to represent biologically distinct entities until replicated and validated independently. The proposed clinical research pathway can be conceptualized as: Standard clinicopathological assessment → serial D-dimer and relevant multimodal data collection → AI-based contextual analysis → explainable, outcome-specific risk estimate → clinician review → evidence-based clinical assessment or intervention Within this pathway, AI does not replace oncological judgment. Instead, it potentially synthesizes complex longitudinal data that may be difficult to interpret manually. The clinical decision remains with the treating team and should follow established diagnostic and therapeutic standards (64). Before integration into precision oncology, AI-enabled D-dimeromics must demonstrate more than statistical association. Models should undergo external and prospective validation across diverse populations, laboratories, treatment settings, and healthcare systems. They should demonstrate robust calibration, clinically meaningful incremental value over existing models, fairness across relevant patient subgroups, and acceptable rates of false-positive and false-negative classifications. Most importantly, impact studies must determine whether providing D-dimeromic predictions to clinicians actually improves decision-making, patient outcomes, or healthcare efficiency. A technically accurate model that does not change management or improve outcomes has limited precision-oncology value (64). Thus, the proposed integration of AI-enabled D-dimeromics with precision oncology is based on a complementary relationship between tumour-centred precision biomarkers and dynamic host-systemic information. Its potential value for the practicing oncologist lies in refining risk assessment, contextualizing longitudinal biomarker changes, supporting assessment of thrombotic risk, identifying potentially important discordant biological trajectories, and enabling research into risk-adapted care. Nevertheless, these applications remain investigational. The immediate priority is to establish whether D-dimeromic signatures are reproducible, independently informative, externally valid, and clinically actionable before they are incorporated into routine breast cancer management (57) (**Figure 3**).

## Challenges and considerations for the clinical implementation of AI-enabled D-dimeromics

Despite its considerable promise, the clinical implementation of AI-enabled D-dimeromics faces several scientific, technical, regulatory, and operational challenges that must be addressed before it can be integrated into routine breast cancer care. Whilst advances in artificial intelligence have substantially improved predictive analytics in oncology, the successful translation of AI-enabled D-dimeromics requires rigorous validation, standardized data acquisition, transparent algorithm development, and careful regulatory oversight. A balanced appraisal of these limitations is essential to ensure that the proposed framework is interpreted as a future precision oncology strategy rather than an immediately deployable clinical tool (22). One of the most significant barriers is the limited availability of prospective external validation. Most published AI models in oncology have been developed using retrospective, single-centre datasets with relatively homogeneous patient populations. Such models often perform well during internal validation but may exhibit reduced accuracy when applied to independent cohorts with different demographic characteristics, disease biology, laboratory practices, imaging protocols, and treatment strategies. Prospective multicentre studies involving geographically and ethnically diverse populations are therefore essential to establish the robustness, reproducibility, calibration, and generalizability of AI-enabled D-dimeromics before clinical adoption (58).

Another major challenge involves data heterogeneity. AI-enabled D-dimeromics relies on integrating diverse data modalities, including longitudinal D-dimer measurements, coagulation biomarkers, genomic profiles, transcriptomic signatures, radiomic features, digital pathology, liquid biopsy results, and electronic health records. These datasets are frequently generated using different laboratory assays, sequencing platforms, imaging acquisition protocols, and institutional workflows, resulting in considerable variability. Furthermore, differences in D-dimer assay methodologies, calibration standards, reporting units, and reference intervals may compromise model portability across healthcare systems. Harmonized laboratory protocols, standardized reporting practices, and interoperable data infrastructures will therefore be critical for developing reliable and transferable predictive models (27). Algorithmic bias represents another important concern. AI systems trained on datasets that underrepresent specific demographic groups, molecular subtypes, or healthcare settings may inadvertently generate unequal predictive performance across populations. Bias may arise from imbalanced datasets, incomplete clinical information, differences in access to healthcare, or variations in treatment practices, potentially contributing to disparities in clinical decision-making. To minimize these risks, future AI-enabled D-dimeromics models should incorporate representative multicentre datasets, fairness-aware machine learning approaches, bias detection algorithms, and continuous performance monitoring across diverse patient populations. Transparent reporting of model development and subgroup performance will also be essential to promote equitable implementation (59).

The interpretability of AI algorithms remains another important consideration. Although complex deep learning models often achieve excellent predictive accuracy, their “black-box” nature may reduce clinician confidence and hinder integration into multidisciplinary oncology practice. Incorporating explainable artificial intelligence (XAI) techniques—including SHapley Additive exPlanations (SHAP), Local Interpretable Model-Agnostic Explanations (LIME), feature importance analyses, and attention-based visualization—can improve transparency by identifying the clinical, laboratory, molecular, and imaging variables contributing to individualized predictions. Enhanced interpretability is likely to facilitate clinician acceptance, patient trust, and regulatory approval (60). Additional technical challenges include missing data, class imbalance, temporal variability, and model drift. Longitudinal oncology datasets frequently contain incomplete laboratory measurements, irregular follow-up intervals, and evolving treatment regimens, all of which can influence predictive performance. Moreover, AI models may experience declining accuracy over time as diagnostic technologies, therapeutic strategies, and patient characteristics evolve. Continuous model updating, periodic recalibration, and learning health-system approaches will therefore be necessary to maintain long-term reliability (21). Regulatory and governance considerations also represent critical components of clinical translation. AI-enabled D-dimeromics would likely be regulated as software supporting clinical decision-making and must therefore satisfy requirements for analytical validity, clinical validity, safety, cybersecurity, transparency, and post-deployment monitoring. Compliance with internationally recognized reporting and evaluation frameworks—including TRIPOD-AI, CONSORT-AI, SPIRIT-AI, and emerging regulatory guidance from health authorities—will improve methodological quality, reproducibility, and confidence in AI-assisted clinical applications. Establishing standardized validation pathways and independent performance auditing will be essential before routine implementation (61).

Furthermore, the integration of AI-enabled D-dimeromics into clinical practice raises important ethical and data governance considerations, including patient privacy, informed consent, secure data sharing, algorithm accountability, and protection against unauthorized access to sensitive health information. Privacy-preserving approaches such as federated learning, secure multiparty computation, and robust data encryption may facilitate collaborative model development whilst maintaining patient confidentiality. Clear governance frameworks defining responsibility for AI-assisted clinical decisions will also be necessary to support safe implementation (62). Practical implementation within healthcare systems presents additional challenges. Successful adoption will require seamless integration with electronic health records, standardized laboratory information systems, digital pathology platforms, and radiology workflows without increasing clinician workload. Adequate computational infrastructure, interdisciplinary collaboration amongst oncologists, haematologists, pathologists, laboratory scientists, radiologists, bioinformaticians, and AI specialists, together with clinician education and institutional support, will be essential for effective deployment.

## Recommendations and future research priorities

To facilitate the translation of AI-enabled D-dimeromics from a conceptual framework to a clinically applicable precision oncology tool, coordinated multidisciplinary research efforts should be prioritized. The immediate focus should be on establishing standardized protocols for D-dimer measurement, including harmonization of assay methodologies, reporting units, quality assurance procedures, and clinically relevant sampling time points. Such standardization is essential to minimize inter-laboratory variability and ensure reproducibility across studies and healthcare settings. A second priority is the development of large-scale prospective multicentre cohorts comprising well-characterized breast cancer populations with standardized clinical, pathological, imaging, molecular, and longitudinal follow-up data. These studies should encompass diverse molecular subtypes, disease stages, treatment regimens, and demographic groups to ensure that AI models are generalizable and equitable across different populations. Serial D-dimer measurements should be incorporated into these protocols to evaluate temporal changes in relation to disease progression, treatment response, recurrence, thromboembolic events, and survival. Parallel efforts should focus on the creation of multimodal D-dimeromics datasets integrating coagulation biomarkers with radiomics, digital pathology, genomics, transcriptomics, proteomics, metabolomics, circulating tumour DNA (ctDNA), and electronic health records. Such comprehensive datasets will provide the foundation for developing robust machine learning and deep learning models capable of identifying complex biological interactions that cannot be detected using conventional statistical approaches.

Future investigations should also prioritize the development of transparent and explainable artificial intelligence (XAI) systems that provide interpretable predictions to support clinician confidence and facilitate regulatory approval. External validation across independent institutions, calibration of prediction models, and assessment of algorithmic fairness should become routine components of model development to minimize bias and improve clinical reliability. A practical research roadmap may include several progressive milestones. Within the next three years, collaborative groups should establish standardized D-dimer collection protocols and initiate prospective multicentre observational studies with predefined patient characteristics and harmonized outcome measures. Within three to five years, externally validated AI-enabled D-dimeromics models integrating clinical, laboratory, imaging, and molecular data should be developed and evaluated in independent populations. Beyond five years, randomized clinical implementation studies should determine whether AI-guided D-dimeromics improves risk stratification, therapeutic decision-making, thromboprophylaxis, surveillance strategies, and patient outcomes compared with current standards of care. Finally, successful clinical translation will require close collaboration amongst oncologists, haematologists, laboratory scientists, radiologists, pathologists, data scientists, bioinformaticians, regulatory agencies, and healthcare policymakers. International data-sharing initiatives, federated learning frameworks, and standardized reporting guidelines should be encouraged to accelerate validation whilst maintaining patient privacy and data security. Through these coordinated efforts, AI-enabled D-dimeromics has the potential to evolve from a promising research paradigm into a clinically validated precision medicine platform capable of identifying ultra-high-risk breast cancer phenotypes and supporting individualized patient management.

## Conclusion

AI-enabled D-dimeromics represents a hypothesis-driven and emerging framework, rather than an established clinical tool, for investigating whether longitudinal D-dimer patterns can improve risk assessment in breast cancer. Its potential value lies in integrating D-dimer kinetics with tumour characteristics, thromboinflammatory biomarkers, comorbidities, treatment exposure, imaging, and other clinical data to identify dynamic risk phenotypes that may not be fully captured by conventional prognostic systems. However, substantial limitations remain, including the nonspecificity of D-dimer, assay and threshold heterogeneity, confounding by age, infection, thrombosis, organ dysfunction and treatment, inconsistent longitudinal sampling, retrospective data bias, algorithmic overfitting, limited interpretability, and the current absence of prospectively validated AI-driven D-dimeromics models in breast cancer. The proposed concept of an ultra-high-risk phenotype likewise requires objective, outcome-specific definitions and external validation before clinical application. Future research should prioritize standardized D-dimer measurement, prospective multicentre longitudinal datasets, transparent and explainable AI modelling, rigorous adjustment for confounders, comparison with established prognostic models, and external and prospective validation. Studies should also determine whether D-dimeromics provides meaningful incremental predictive value and, ultimately, improves clinical decision-making and patient outcomes. Until these requirements are met, AI-enabled D-dimeromics should remain a research framework for testing whether dynamic thrombo-fibrinolytic signatures can contribute meaningfully to precision breast oncology.
